# Evaluation of early postoperative intravenous opioid rescue as a novel quality measure in patients who receive thoracic epidural analgesia: a retrospective cohort analysis and prospective performance improvement intervention

**DOI:** 10.1186/s12871-021-01332-7

**Published:** 2021-04-19

**Authors:** Nadav Levy, Peter Santer, Liana Zucco, Sarah Nabel, Galina Korsunsky, Satya Krishna Ramachandran

**Affiliations:** Department of Anesthesia, Critical Care and Pain Medicine, Beth Israel Deaconess Medical Center, 330 Brookline Ave, Boston, MA 02215 USA

**Keywords:** Thoracic epidural analgesia, Regional anesthesia, Efficiency metrics, Quality improvement, Perioperative analgesia

## Abstract

**Background:**

In this study, we explored the utility of intravenous opioid rescue analgesia in the post anesthesia care unit (PACU-OpResc) as a single marker of thoracic epidural analgesia (TEA) failure and evaluated the resource implications and quality improvement applications of this measure.

**Methods:**

We performed a retrospective analysis of all TEA placements over a three-year period at a single academic medical center in Boston, Massachusetts. The study exposure was PACU-OpResc. Primary outcome was PACU length of stay (LOS). Secondary outcomes included reasons for delayed PACU discharge and intraoperative hypotension. The analyses were adjusted for confounding variables including patient comorbidities, surgical complexity, intraoperative intravenous opioids, chronic opioid use and local anesthetic bolus through TEA catheter. Post analysis chart review was conducted to determine the positive predictive value (PPV) of PACU-OpResc for inadequate TEA. As a first Plan-Do-Study-Act cycle, we then introduced a checkbox for documentation of a sensory level check after TEA placement. Post implementation data was collected for 7 months.

**Results:**

PACU-OpResc was required by 211 (22.1%) patients who received preoperative TEA, was associated with longer PACU LOS (incidence rate ratio 1.20, 95% CI:1.07–1.34, *p* = 0.001) and delayed discharge due to inadequate pain control (odds ratio 5.15, 95% CI 3.51–7.57, *p* <  0.001). PACU-OpResc had a PPV of 76.3 and 60.4% for re-evaluation and manipulation of the TEA catheter in PACU, respectively. Following implementation of a checkbox, average monthly compliance with documented sensory level check after TEA placement was noted to be 39.7%. During this time, a reduction of 8.2% in the rate of PACU-OpResc was observed.

**Conclusions:**

This study demonstrates that PACU-OpResc can be used as a quality assurance measure or surrogate for TEA efficacy, to track performance and monitor innovation efforts aimed at improving analgesia, such as our intervention to facilitate sensory level checks and reduced PACU-OpResc.

**Trial registration:**

not applicable.

## Background

Thoracic epidural analgesia (TEA) is a widely accepted and effective modality for postoperative pain management after thoracic and abdominal surgery [1, 2]. The specific benefits of TEA include better quality of pain control, decreased incidence of respiratory complications and reduced postoperative nausea and vomiting when compared to parenteral opioids [1, 3–6]. Thoracic epidural analgesia has a reported failure rate of up to 32% [7], but the definition of ‘failure’ varies significantly in the literature [8]. Several factors can be attributed to thoracic epidural failure, including technical (catheter placement, equipment), patient-related (difficult anatomy) or pharmacological (drugs and doses) causes [9, 10]. Furthermore, the placement and management of TEA is complex from a work-flow perspective and requires time, resources and expertise across several domains of care. The high risk of failure, considering the potential benefit to surgical patients, makes TEA a high-yield patient-centered care area for performance improvement.

Traditionally, quality improvement efforts have focused on methods to streamline the TEA placement process, limit delays related to TEA placement on the overall operating room workflow and improve compliance with intraoperative epidural infusion [9–13]. While most of these improvement measures address local departmental issues, there are limited data on TEA surveillance at a system-level. We therefore conducted a retrospective observational study to explore the prevalence of intravenous opioid rescue analgesia in the postanesthesia care unit (PACU-OpResc) in patients with TEA and evaluated the feasibility of this measure as a marker of TEA failure.

## Methods

### Aim

We hypothesized that patients with TEA requiring postoperative rescue opioids in the PACU experienced prolonged lengths of stay in the PACU due to inadequate pain control. Our aim therefore was to evaluate the use of PACU-OpResc, as a patient centered metric for surveillance of TEA effectiveness, to pilot an intervention to target improvement in this patient population.

Furthermore, demonstrating a sensory block after a local anesthetic bolus is a common and recommended method to verify correct placement of TEA catheter [8, 10, 12] that may reduce or aid in resolving cases of improper TEA placements. However, due to time constrains, this is often omitted preoperatively or performed postoperatively [12, 14]. Therefore in the second phase of the study, we hypothesized that an intervention to encourage the performance and recording of a sensory level check after TEA placements may reduce PACU-OpResc rates.

### Setting

Eligible patients undergoing thoracic or major abdominal surgery at Beth Israel Deaconess Medical Center (BIDMC), Boston, Massachusetts are routinely offered TEA for intra/postoperative pain management. Approximately 400 TEAs are placed preoperatively at BIDMC yearly. Thoracic epidurals are sited either by the anesthesiologist assigned to the case or by a member of the Acute Pain Service (APS), pending availability. The APS is a designated team of anesthesiologists and rotating residents who are also responsible for the postoperative management of all TEAs. Placement of TEAs is routinely performed in the preoperative holding area, under standard monitoring, using a FlexTip Plus® epidural catheterization kit with a 17 g Touhy needle (Teleflex, Chelmsford, USA). The position (sitting or lateral decubitus), approach (midline/ para-median) and the loss of resistance technique (saline/ air) are at the discretion of the provider. A test dose of lidocaine (1%) with epinephrine (1:100,000) in approximately 3 mL, which is included in the catheterization kit, is routinely administered into the catheter and charted. The patient is then taken to the operating room where general anesthesia is induced. A preprepared epidural infusion solution (0.1% Bupivacaine with 10 mcg/ml of Hydromorphone) is available during the intraoperative period and is administered via a designated epidural pump and extension set (CADD®- Solis, Smiths Medical, Dublin, USA). The start time, epidural infusion rate, bolus volume and bolus frequency are at the discretion of the anesthesiologist assigned for the case. Upon arrival to the PACU, postoperative analgesic care is managed by trained nursing staff, who consult the APS team if any issues regarding TEA arise.

In this study we report, in phase 1, a retrospective review of all surgical cases performed at BIDMC from January 1st 2016 to December 31st 2018 that were documented in the electronic health record. This established the feasibility of PACU-OpResc as a metric which our organization began tracking monthly, as of January 1st 2019. Phase 2 of our study describes a quality improvement initiative involving the documentation of a sensory level check (SLC) and includes the PACU-OpResc rates as measured throughout phase 1 and up to April 30th 2019 (preintervention) and a 7 month period postintervention (May 1st 2019 to November 30th, 2019).

### Phase 1: retrospective cohort analysis

#### Study design

Data collected for each patient included age, body mass index (BMI), the duration and type of surgical procedure and study specific intra and postoperative variables described below. Surgical complexity was defined by the work relative value units (work-RVUs) for each case. Intraoperative and postoperative data included the administration of IV opioids (morphine equivalents), adjunct analgesic medications, local anesthetic boluses and infusions via the TEA. Opioid prescriptions during the 90 days prior to surgery were reviewed as a way to identify patients at higher risk of PACU delays due to pain.

#### Study population

The study population included all surgical patients admitted to our center between January 1st 2016 to December 31st 2018 for whom a TEA catheter was placed prior to a surgical procedure. Patients directly admitted to an intensive care unit from the operating room without intermediate stay in the PACU were excluded. Patients with missing data for confounder variables were excluded for complete-case analysis.

#### Exposure

The exposure was defined as a binary variable indicating any requirement for IV opioid administration during the PACU stay.

#### Outcomes

The primary outcome was defined as PACU length of stay (LOS) in minutes. Secondary outcomes included PACU delay, defined as ineligibility for discharge after 3 h of stay and the reason for such a delay. In our center, delays in PACU are automatically registered in the electronic medical records and trigger a forced action, requiring the PACU nurse to document the reason of the delay by selecting it from a predefined dropdown list. Secondary outcomes also included measures of intraoperative hemodynamic stability as reflected by low blood pressure values, defined by frequency and duration of mean blood pressure below 65 mmHg and 55 mmHg as recorded in the electronic anesthesia chart.

#### Covariate model

Based on clinical plausibility and available literature, analyses were adjusted for the following patient-specific covariates: age, BMI, 90-day preoperative opioid prescription, and history of drug abuse. Case specific confounders included the duration of surgery, work relative value units (RVU), total dose of intraoperatively administered opioids (morphine equivalents), local anesthetic TEA boluses, TEA infusion duration and a high risk of postoperative pulmonary complications (i.e. Score for Prediction of Postoperative Respiratory Complications [SPORC] ≥7) [15].

#### Statistical analyses

Negative binomial regression was used to assess the association between the dichotomized exposure and PACU length of stay as well as duration of hypotension. For binary endpoints, we employed multivariable logistic regression. Statistical significance was assumed at a *P* value < 0.05 for the primary analysis. Statistical analyses were performed using Stata (version 15; StataCorp LLC, College Station, TX).

We conducted a post analysis chart review of all APS notes for the exposure group to estimate the sensitivity of our measure. We calculated the positive predictive value of PACU-OpResc for documented APS consultation during PACU stay and for any documented intervention to improve analgesia (e.g. epidural catheter manipulation, replacement, bolus, additional IV medication or opioid infusion).

### Phase 2: performance improvement intervention

#### Intervention implementation

We implemented an intervention using the Model for Improvement [16] and through a structured, first Plan-Do-Study-Act (PDSA) cycle, we introduced a sensory level check (SLC) discrete binary documentation element in the electronic procedure note (Compurecord, Phillips Healthcare, Andover, MA, USA). Marking this checkbox prompted another binary checkbox in which to document whether an adequate, bilateral sensory band was achieved after TEA placement. The SLC checkbox was added to the digital procedure chart on May 1st 2019, and its implementation was communicated to the APS team in a formal presentation, introducing the documentation change. Our intervention study measures were defined as SLC documentation (process measure) and PACU-OpResc (outcome measure). We collected post implementation data (intervention study measures) from May 1st to November 30th 2019. Post intervention PACU-OpResc Rates were compared to preintervention rates, as collected from January 1st 2016 to April 30th 2019.

#### Statistical analysis

A 3-sigma statistical process control “P” chart was used to monitor and analyze the monthly average PACU-OpResc exposure. Rates of PACU-OpResc exposure were compared using a Likelihood ratio ChiSquare test. Data for the performance improvement study data were analyzed using JMP®Pro (Version 14.0.0. SAS Institute Inc., Cary, NC,1989–2019). This manuscript adheres to the applicable STROBE [17] and SQUIRE [18] checklists (Phase 1 and Phase 2, respectively).

## Results

### Phase 1: retrospective cohort analysis

#### Study cohort characteristics

**Fig. 1:**
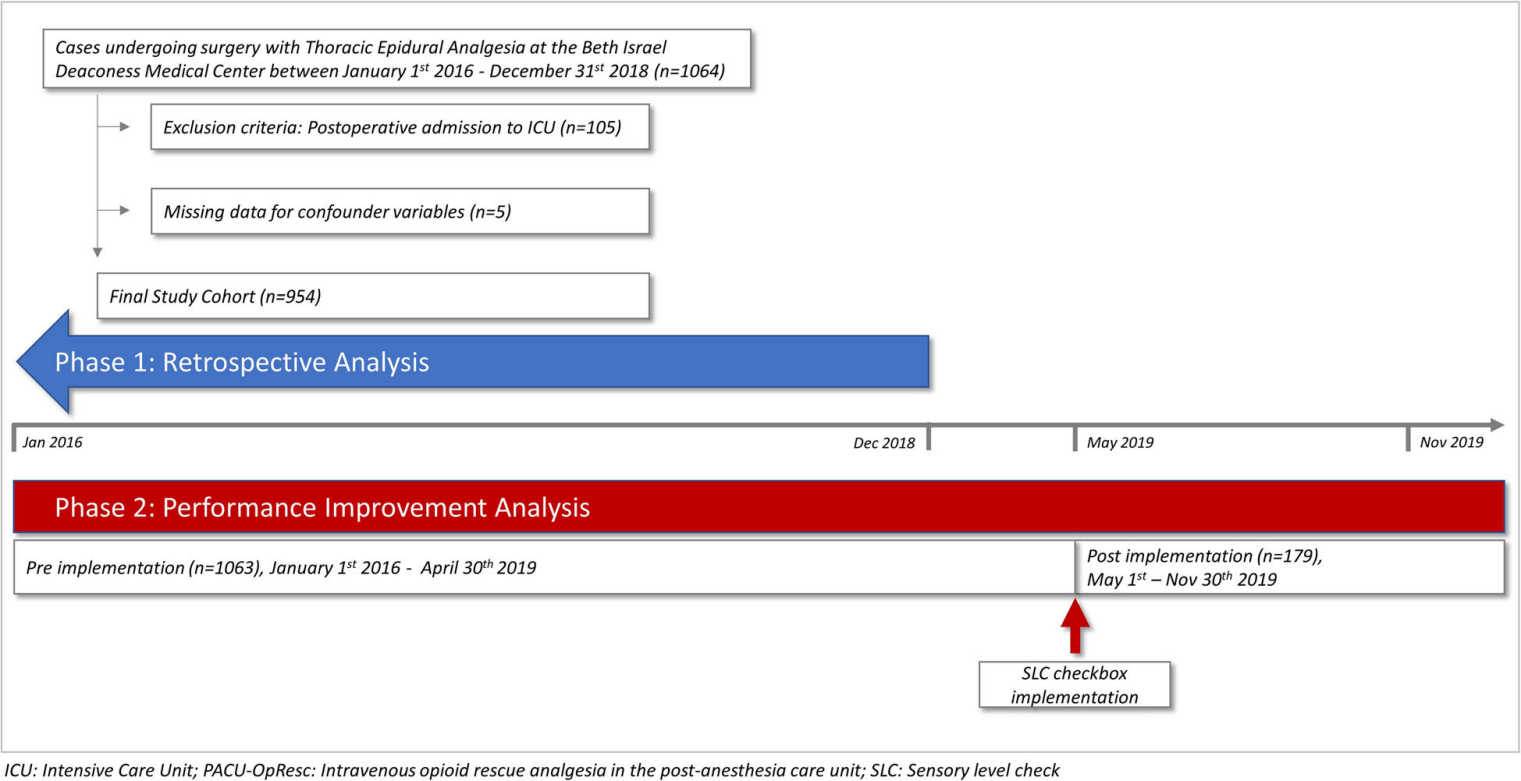
Study flow diagram: Flow diagram of the retrospective analysis of Thoracic Epidural Placements over a three-year period (Phase 1). Outcomes of this study were used to plan the performance improvement study (Phase 2), where pre and post intervention monthly rates of PACU-OpResc were compared

A total of 1064 TEA catheters were placed for surgical patients at our center during the study period.. After excluding 105 patients who were admitted directly to intensive care units and 5 patients for whom data was missing for confounder variables, our final study cohort included 954 TEA placements (Fig. 1). Among patients with preoperative TEA, 211 (22.1%) were exposed to PACU-OpResc. The study population characteristics and procedural data (TEA placement) are presented in Table 1.

**Table 1:** Baseline characteristics of study population

Characteristic		No PACU-OpResc (***n*** = 743)	PACU-OpResc (***n*** = 211)
Age (years)		62.00 ± 13.34	58.93 ± 12.92
Sex	Male	321 (43.2%)	97 (46.0%)
	Female	422 (56.8%)	114 (54.0%)
Height, cm		167.77 ± 10.08	168.16 ± 10.12
Weight, kg		79.78 ± 22.15	77.66 ± 19.28
BMI, kg/m^2^		28.20 ± 6.91	27.39 ± 6.03
ASA Status	1	14 (1.9%)	1 (0.5%)
	2	218 (29.3%)	58 (27.5%)
	3	483 (65.0%)	143 (67.8%)
	4	28 (3.8%)	9 (4.3%)
Surgical Service	General	409 (55.0%)	120 (56.9%)
	Gynecology	146 (19.7%)	28 (13.3%)
	Not documented	81 (10.9%)	17 (8.1%)
	Thoracic	77 (10.4%)	38 (18.0%)
	Vascular	21 (2.8%)	6 (2.8%)
	Orthopedics	5 (0.7%)	1 (0.5%)
	Plastics	4 (0.5%)	1 (0.5%)
Duration of surgery, min		267.00 (184.00, 393.00)	258.00 (188.00, 384.00)
Work RVUs		28.78 ± 14.48	27.71 ± 13.48
High SPORC score		26 (3.5%)	5 (2.4%)
Preoperative opioid prescription*		127 (17.1%)	75 (35.5%)
Intraoperative morphine equivalent dose (mg)		12.00 (10.00, 20.00)	15.00 (10.00, 25.00)
PACU morphine equivalent dose (mg)		0.00 (0.00, 0.00)	5.00 (2.50, 7.50)
TEA Placement and management			
Placement in sitting position		640 (86.1%)	177 (83.9%)
Midline Approach		322 (43.3%)	87 (41.2%)
Documented local anesthetic bolus		182 (24%)	66 (31%)
First Case		361 (48.6%)	100 (47.4%)
Placement Attempts	1	402 (54.1%)	103 (48.8%)
	2	163 (21.9%)	45 (21.3%)
	≥3	88 (11.8%)	38 (17.9%)
	Not documented	90 (12.1%)	25 (11.8%)
Placement duration, min		13.00 ± 8.30	13.72 ± 9.45
Time from surgical Incision to TEA infusion start (minutes)		27.60 ± 71.74	31.83 ± 74.59
Epidural infusion during > 50% of case time		603 (81.2%)	146 (69.2%)

Data are expressed as mean ± standard deviation, frequency (prevalence in %) or median (interquartile range (25th–75th percentile), values separated by comma). *Opioid prescription within 90 days prior to surgery

*ASA* American Society of Anesthesiologist; *BMI* Body Mass Index; *PACU* Post Anesthesia Care Unit; *PACU-OpResc* intravenous opioid rescue analgesia in the post anesthesia care unit; *RVUs* Relative Value Units; *SPORC* Score of Prediction of postOperative Respiratory complications; *TEA* Thoracic Epidural Analgesia;

#### Primary outcome

In patients exposed to PACU-OpResc, the median (IQR) PACU LOS was 286 (221, 427) min compared to 269 (194, 381) min in patients who did not require PACU-OpResc. After adjusting for prespecified confounders (listed under Covariate Model), PACU-OpResc was associated with prolonged PACU length of stay (adjusted incidence rate ratio (aIRR) 1.20, 95% CI 1.07–1.34, *p* = 0.001), corresponding to an adjusted absolute difference of 76.1 min (95% CI 27.3–125.0 min).

#### Secondary outcomes

A total of 128 (60.66%) patients exposed to PACU-OpResc experienced PACU delays for any reason compared to 283 (38.09%) delays in patients not requiring rescue opioids. Exposure to PACU-OpResc was associated with overall delay of discharge from the PACU (adjusted odds ratio (aOR) 2.57, 95% CI 1.83–3.60, *p* <  0.001). PACU discharge delays due to pain occurred more often in patients exposed to PACU-OpResc: 41.23% of patients in the PACU-OpResc group were delayed due to pain compared to only 12.11% in the no PACU-OpResc group. The need for rescue opioids was associated with pain-related PACU discharge delays (aOR 5.15, 95% CI 3.51–7.57, *p* <  0.001). Exposure to PACU-OpResc was not associated with hemodynamic compromise as expressed by the duration of intraoperative hypotension, defined as a mean arterial pressure below 65 or 55 mmHg. Outcome data are presented in Table 2.

**Table 2:** Association of PACU-OpResc with primary and secondary endpoints

Outcomes	No PACU-OpResc (***n*** = 743)	PACU-OpResc (***n*** = 211)	Unadjusted analysis		Adjusted analysis	
			OR/IRR (95% CI)	***p***-value	OR/IRR (95% CI)	***p***-value
**Primary Outcome**						
PACU length of stay, min	269 (194, 381)	286 (221, 427)	1.21 (1.08, 1.35)	0.001	1.20 (1.07, 1.34)	**0.001**
**Secondary Outcomes**						
PACU discharge delay						
Any reason, n (%)	283 (38.09%)	128 (60.66%)	2.51 (1.83, 3.43)	< 0.001	2.57 (1.83, 3.60)	**< 0.001**
Pain, n (%)	90 (12.11%)	87 (41.23%)	5.09 (3.58, 7.24)	< 0.001	5.15 (3.51, 7.57)	**< 0.001**
Cardiovascular, n (%)	125 (16.82%)	42 (19.91%)	1.23 (0.83, 1.81)	0.299	1.34 (0.88, 2.04)	0.175
PONV, n (%)	5 (0.67%)	3 (1.42%)	2.13 (0.50, 8.98)	0.304	1.76 (0.34, 8.96)	0.498
Sedation/Respiratory, n (%)	65 (8.75%)	22 (10.43%)	1.21 (0.73, 2.02)	0.455	1.01 (0.57, 1.80)	0.978
Voiding, n (%)	51 (6.86%)	23 (10.90%)	1.66 (0.99, 2.79)	0.055	1.63 (0.93, 2.85)	0.085
Hypotension MAP < 65 mmHg						
Frequency, n (%)	710 (95.56%)	201 (95.26%)	0.93 (0.45, 1.93)	0.854	1.04 (0.47, 2.31)	0.924
Duration, min	16 (6, 32)	14 (6, 28)	0.84 (0.71, 1.00)	0.048	0.96 (0.82, 1.12)	0.587
Hypotension MAP < 55 mmHg						
Frequency, n (%)	485 (65.28%)	128 (60.66%)	0.82 (0.60, 1.12)	0.218	0.90 (0.64, 1.26)	0.531
Duration, min	2 (0, 5)	1 (0, 5)	0.85 (0.66, 1.08)	0.184	0.94 (0.74, 1.21)	0.651

Data are expressed as frequency (prevalence in %) or median (interquartile range (25th–75th percentile), values separated by comma). Statistical analyses were performed using negative binomial regression (PACU length of stay, duration of hypotension) or multivariable logistic regression (all other analyses). Odds ratios (OR) are reported for logistic regression analyses, incidence rate ratios (IRR) for negative binomial regression analyses

*MAP* Mean Arterial Pressure; *PACU* Post Anesthesia Care Unit; *PACU-OpResc* intravenous opioid rescue analgesia in the postanesthesia care unit; *PONV* Post-Operative Nausea and vomiting

In a post analysis review of the APS notes for all patients exposed to PACU-OpResc (*n* = 211), we found 168 documented APS evaluations in PACU and 128 documented PACU interventions to improve pain management. This represents a positive predictive value (PPV) of PACU-OpResc for APS evaluation and intervention of 76.3 and 60.4%, respectively.

### Phase 2: intervention study

**Fig. 2:**
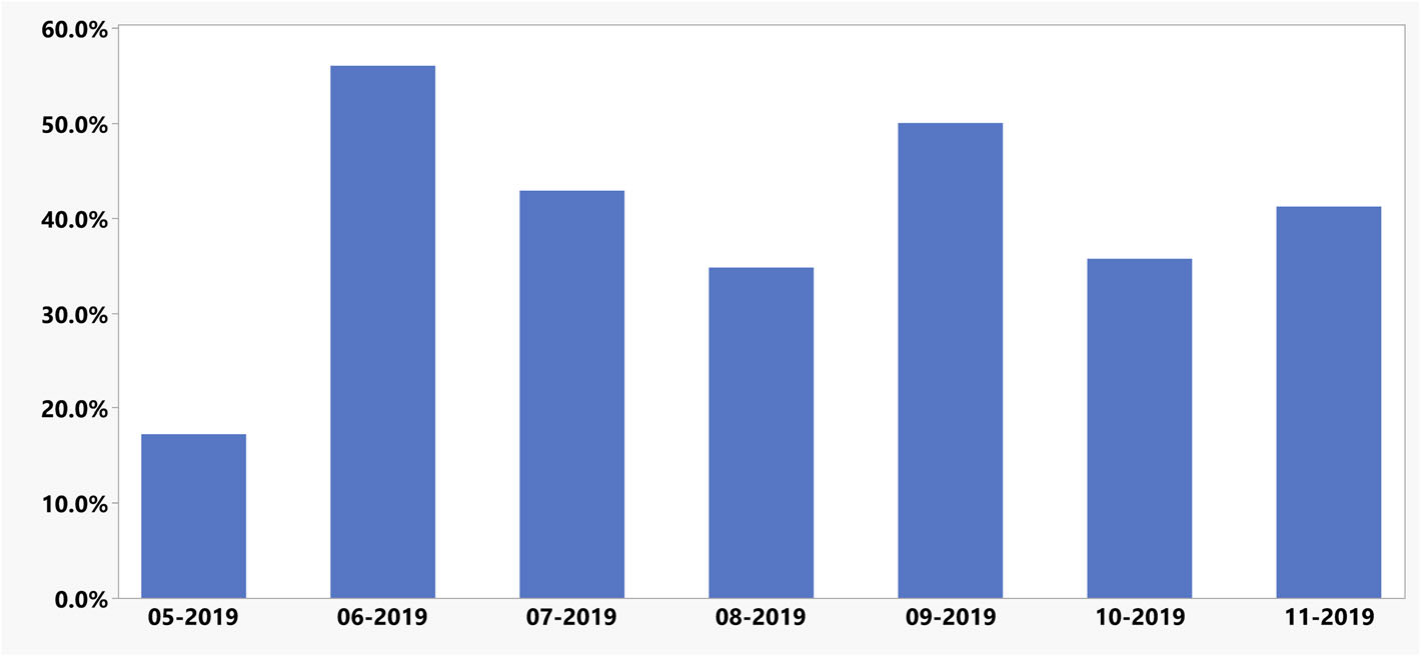
Monthly Rate of Thoracic Epidurals with Documented Sensory Level Check. Percentage of Thoracic Epidurals with documented sensory level check following implementation of discreate SLC documentation field in the electronic procedure note. Data presented as % [SLC] /[Total TEA placements] per month

**Fig. 3:**
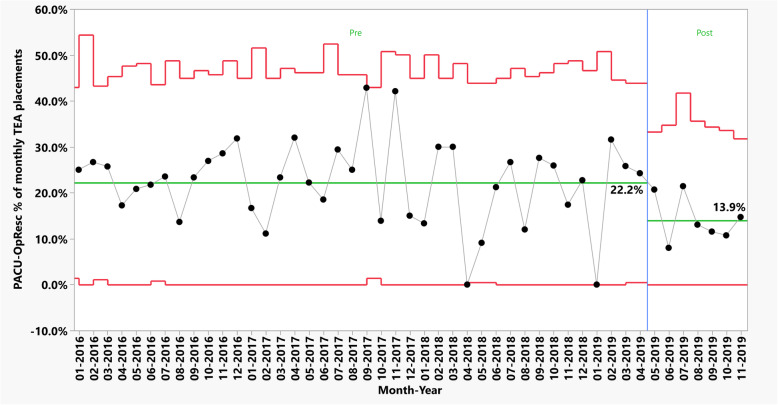
Phased process control chart of monthly PACU-OpResc. Phased statistical process control chart (p-chart) of PACU-OpResc as a proportion of monthly TEA. Middle horizontal line reflects the weighted average PACU-OpResc rate before (22.2%) and after (13.9%) implementing SLC documentation. Upper and lower horizontal lines reflect the upper and lower 3σ control limits for each month, respectively. Vertical line marks the implementation of SLC documentation (May 2019). Sample size (TEA placements) was insufficient to determine the lower control limit during April 2018 and January 2019, therefore the zero-PACU-OpResc rate in these months is not considered to be “special cause”. PACU: Post Anesthesia Care unit; PACU-OpResc: Intravenous opioid rescue analgesia in the post-anesthesia care unit; SLC: Sensory level check; TEA: Thoracic Epidural Analgesia

In the post intervention phase, 179 TEAs were placed preoperatively for surgical patients. The mean ± SD placement rate remained consistent at 26.6 ± 5.4 and 25.6 ± 6.2 TEAs per month, across pre and post intervention periods, respectively. The average compliance with SLC documentation was 39.7% for the entire post intervention phase. Compliance per month is presented in Fig. 2. A prepost analysis demonstrated an absolute decrease of 8.2% in the average exposure to PACU-OpResc during the post implementation phase when compared to the preceding years (Table 3). Plotting the data using a 3-sigma process control chart, we found no special causes and outliers during the post intervention period, further supporting our findings (Fig. 3).

**Table 3:** PACU-OpResc rates before and after implementing sensory level check documentation

	PACU-OpResc		
	No	Yes	Total
**Before SLC implementation (January 2016–April 2019)**	827 (77.80%)	236 (22.20%)	1063
**After SLC implementation (May–November 2019)**	154 (86.03%)	25 (13.97%)	179
**Total**	981	261	1242

Data analyzed using a likelihood ratio ChiSquare test (p-value = 0.009). PACU-OpResc: intravenous opioid rescue analgesia in the post anesthesia care unit; SLC: Sensory level check

## Discussion

In this study, we found exposure to PACU-OpResc to be associated with prolonged PACU length of stay and delayed PACU discharge. We then demonstrated the utilization of this metric in a quality improvement intervention focusing on TEA catheter placement verification. Thoracic epidural analgesia is a widely used modality for intra/postoperative pain management in major abdominal and thoracic surgeries, recommended by enhanced recovery after surgery (ERAS) protocols for several surgical disciplines [19–21]. Thus, when offered to patients preoperatively, TEA is often presented as a superior modality to IV analgesia that may contribute to faster recovery, better overall experience and perhaps improved outcome. Our findings imply that for a substantial number of patients PACU-OpResc represents inadequate analgesia requiring expert intervention.

### Phase 1: patient centered measure for Thoracic Epidural Analgesia effectiveness

The primary outcome of this study was chosen to reflect the effect of inadequate TEA on resource allocation. Taking into account that inadequate pain management is a known factor for increased PACU length of stay [22], we theorized that exposure to PACU-OpResc, as a surrogate for inadequate TEA will prolong PACU length of stay. Indeed, our analysis, adjusted for patient and procedure specific confounders, showed that patients who required PACU-OpResc had longer PACU length of stay. It is difficult to assess the clinical impact and significance of such a delay, however, for most of our study population, the prolonged PACU stay was due to inadequate pain management. We, therefore, believe that this then becomes clinically significant. The operational burden of patients delayed in PACU can be quantified and reflected in the availability of nurses and the potential effect OR turnover. For most of the patients in our cohort, the pain management in PACU required the involvement of a multidisciplinary team, an effort that may have been avoided if adequate TEA is provided. We suggest that investing resources to verify adequate TEA and catheter placement, even though it may delay the OR workflow, may prove to be cost-effective, reduce PACU length of stay for these patients.

The definition of failed epidural analgesia varies substantially between authors and ranges from specific criteria such as failure to place a catheter at 1st attempt [23] to broader definitions which include “documented inability to locate the epidural space during insertion or complete lack of any surgical site sensory block following epidural bolus” [12] or “any condition during the course of treatment that requires epidural catheter replacement or the addition of another major treatment modality such as IV patient- controlled analgesia” [7]. Irrespective of nomenclature, failure to achieve adequate analgesia requiring rescue analgesic management in the immediate postoperative phase represents an undesirable outcome for the patient and could be considered a marker of TEA failure. In planning this study and intervention, we focused on establishing a framework for a learning environment that would prospectively enable feedback on the effectiveness of staged quality improvement interventions. We chose to focus on PACU-OpResc as a pragmatic measure that represents the overall patient experience, regardless of the reason for inadequate TEA.

The rate of PACU-OpResc of 22.1% in our center lies within the previously reported range of TEA failure rates and supports PACU-OpResc as a surrogate measure for inadequate TEA. We further demonstrated good positive predictive value of PACU-OpResc for documented evaluation by the APS team and documented interventions to improve TEA effectiveness in PACU. Evaluation of patients with TEA and interventions may be performed by anesthesiology teams in the PACU or the OR and may not always be documented. We, therefore, assume that our calculated PPV is an underestimation of the ability of PACU-OpResc to identify cases of inadequate TEA.

### Phase 2: intervention study

While mapping and assessing the TEA placement process for potential points of intervention, we noted that on perceived successful placement of a TEA and following the administration of a standardized test dose, a sensory level check (SLC) was not being consistently performed. Furthermore, if a SLC was performed it was not documented due to the lack of a discrete documentation element in the electronic procedure note. We focused on the SLC documentation as a first PDSA cycle, through which we could gain knowledge both on the TEA process and PACU-OpResc as its measure. Our aim was to assess firstly, compliance with SLC documentation immediately after TEA placement and secondly, the impact of SLC documentation on TEA failure rates as represented by PACU-OpResc.

The declared goal of our intervention was to verify that the epidural catheter is well-located and provides the desired sensory block. However, in our center, where one team places the TEA and another uses it in the OR, we theorized that taking a patient to the OR with a proven-to-be-working TEA, may incentivize physicians to utilize it better, which in turn may contribute to the reduction in PACU-OpResc.

Larsson et al. [14] demonstrated that adequate sensory level after a local anesthetic bolus can be achieved in 35% of the patients within 5–6 min, and in 99% of the patients within 15 min. As we were aware of the time constraints and pressure to quickly take patients to the OR, we deliberately did not mandate or actively promote conducting a sensory level check throughout the intervention period. Thus, we believe that a level check was conducted after placement only when OR workflow permitted it, and that our compliance rate of 39.7% with level checking and documenting adequate sensory level after TEA placement, reflects an inherent ability and resources to do so in our center. We speculate that conducting a level check as well as the ability to document it have reduced PACU-OpResc by allowing for timely manipulation of improperly placed catheters and by encouraging the intraoperative infusion of local anesthetics through the TEA catheter by the OR team. We could neither identify alternative explanations or interventions aimed at reducing opioid administration in our PACU nor detect a significant change in our anesthesia faculty, technique or equipment during our implementation period. Trends in current anesthesia practice aimed at reducing perioperative opioid administration [24–26] may have influenced our findings. However, this would explain a gradual reduction in opioid dosing rather than a significant drop in PACU-OpResc.

### Limitations

This study is a single center retrospective analysis followed by an intervention which despite being effective in our center may not be applicable elsewhere. However, administration of opioids in PACU is both pragmatic, objective and feasible metric for TEA effectiveness which may be a measure for local standard of care and management of TEAs. There are, however, biases for using this measure. Inadequate TEA may be overestimated by including patients receiving opioids as part of their postoperative anesthetic plan due to known dependency, or for whom a clinical decision to not use the TEA was made. We attempted to control for this confounder by adjusting our analysis for chronic opioid use. Hypotension, a common reason to discontinue the TEA infusion was included in our secondary outcomes and did not differ between the two study groups.

Underestimation of inadequate TEA using PACU-OpResc may occur when the epidural catheter was replaced, manipulated or used for local anesthetic bolus before a patient received IV opioids, or cases in which adequate analgesia was achieved with non-opioid supplement analgesics. Nevertheless, this adds strength to PACU-OpResc as a measure of the overall process and management of TEAs, also reflecting the timely identification and correction of inadequate TEA to provide better patient care. Our results are further limited by their retrospective nature and by the possibility that factors other than our intervention were responsible for this result, despite our best efforts to eliminate such historical bias.

## Conclusions

The requirement for PACU-OpResc in patients with TEA was shown to prolong PACU stay. These delays were significantly associated with inadequate pain control. This surrogate measure for TEA effectiveness can be used as a metric for ongoing quality improvement projects, reflecting not only the TEA placement but also the management and intervention to improve analgesia by the local team. A simple intervention to verify catheter location was shown to reduce PACU-OpResc in this single center study. Studies to assess the generalizability of this single-center study and effects of further interventions on PACU-OpResc are necessary.

## Data Availability

The data that support the findings of this study are available on request from the corresponding author, [NL]. The data are not publicly available due to their containing information that could compromise the privacy of participants.

## Abbreviations

APS: Acute Pain Service
BMI: Body Mass Index
LOS: Length of Stay
PACU: Post Anesthesia Care Unit
PACU-OpResc: Opioid Rescue in the Post Anesthesia Care Unit
PDSA: Plan-Do-Study-Act
PPV: Positive Predictive Value
RVUs: Relative Value Units
SLC: Sensory Level Check
SPORC: Score of Prediction of postOperative Respiratory complications
TEA: Thoracic Epidural Analgesia
